# Measurement of MMP-7 in micro-volume peripheral blood: development of dried blood spot approach

**DOI:** 10.3389/fped.2023.1293329

**Published:** 2023-11-15

**Authors:** Jingying Jiang, Shuyang Liu, Min Du, Jiale Deng, Gong Chen, Yifan Yang, Rui Dong, Zhuo Fang, Shan Zheng

**Affiliations:** ^1^ Department of Pediatric Surgery, Shanghai Key Laboratory of Birth Defect, and Key Laboratory of Neonatal Disease, Ministry of Health, Children’s Hospital of Fudan University, Shanghai, China; ^2^ Department of Data & Analytics, WuXi Diagnostics Innovation Research Institute, Shanghai, China

**Keywords:** biliary atresia, diagnosis, dried blood spot, MMP-7, screening

## Abstract

**Purpose:**

Serum matrix metalloproteinase-7 (MMP-7) is significant in differentiating biliary atresia (BA). This study aims to develop a new peripheral blood quantitative collection device to detect MMP-7 levels via dried blood spot (DBS).

**Methods:**

This is a diagnostic accuracy test. Serum and DBS MMP-7 concentrations were measured using an ELISA kit. Intraoperative cholangiography and subsequent histological examinations were used to confirm BA diagnoses.

**Results:**

A total of 241 infants with obstructive jaundice were enrolled, among whom 168 were BA. Linear regression showed DBS MMP-7 correlated well with serum MMP-7 (*R* = 0.93, *P* < 0.001). The best cut-off value of serum MMP-7 for BA was 25.9 ng/ml, achieving the area under the ROC curve (AUC) of 0.962 (95% CI: 0.941, 0.983), and the sensitivity, specificity, positive predictive value (PPV), and negative predictive value (NPV) were 86.9%, 94.5%, 97.3% and 75.8%, respectively. The best cut-off value of DBS MMP-7 for BA was 12.5 ng/ml, achieving the AUC of 0.922 (95% CI: 0.888, 0.956), and the sensitivity, specificity, PPV, and NPV were 86.9%, 89.0%, 94.8%, and 74.7%, respectively. The dried blood spots were intervened under different storage conditions, including 1–5 days at room temperature, 2 or 3 days at 30 °C and 2 or 3 days at 37 °C. The DBS MMP-7 concentration under different storage conditions had good correlation and consistency with that at −80 °C.

**Conclusions:**

Serum and DBS MMP-7 correlate well, both of which have high accuracy in the diagnosis of BA, while the requirements for the storage of DBS are low.

## Introduction

Biliary atresia (BA) is a severe liver disease characterized by fibro-inflammatory destruction of bile ducts, resulting in cholestasis and liver fibrosis in neonates and infants (1). Kasai portoenterostomy (KPE) is the consensual treatment strategy to restore bile flow (1–5). To date, it remains difficult to accurately differentiate BA from other cholestatic diseases including Alagille syndrome based on liver function tests or grayscale ultrasound scans, which are the most widely used tests in clinical practice (6–8).

Matrix metalloproteinase-7 (MMP-7) plays an important role in remodeling the extracellular matrix (ECM), which is closely related to liver fibrosis progression (9–11). Recent studies have confirmed the clinical value of serum MMP-7 levels to discriminate BA, which can achieve the area under receiver operating characteristics (ROC) curve of more than 0.9, and both a sensitivity and specificity of between 90% and 95% (12–15).

However, the diagnostic value of MMP-7 expression has been reported on the basis of testing serum samples, which are challenging for sample collection, storage and transportation, and lead to unnecessary blood waste, as only 10–20 µl of serum is required for enzyme-linked immunosorbent assays (ELISAs). An innovative, less invasive method should be developed to further facilitate using MMP-7 levels and prospect its use in neonatal screening.

Thus, we conducted this study to develop an innovative approach to simplify sample collection and transportation that utilizes the dried blood spot (DBS) device and validate its diagnostic accuracy in BA.

## Methods

### Study design

This was a single-center diagnostic test. We developed a novel approach to simplify sample collection that involved collecting heel tip blood and detecting MMP-7 levels using dried blood spot (DBS).

This study was reviewed and approved by the Ethics Committee of Children's Hospital of Fudan University (No.: 2020–296). This work was performed in compliance with the Declaration of Helsinki and other relevant regulations. Informed consent was obtained from the guardian of each participant before enrollment.

### Subjects

This study was conducted at Children's Hospital of Fudan University, an urban tertiary care academic children's hospital in Shanghai, China.

Infants who were admitted for cholestasis were consecutively enrolled. Cholestatic subjects were defined as having a serum direct bilirubin level >17 μmol/L and >20% of total bilirubin.

### Sample collection and storage

Serum and DBS samples were obtained simultaneously after enrollment. DBS samples were collected using quantificational device of 10 µl which was patented by Wuxi Diagnostics. Three duplicates were collected for each patient. One of the DBS samples was transported and stored at room temperature for 1–5 days before measurement, while the rest of DBS samples as well as the serum sample were transported with dry ice and stored at −80 °C.

The DBS samples were intervened under different storage conditions, including 2 or 3 days at 30 °C and 2 or 3 days at 37 °C after removing from −80 °C.

### Measuring MMP-7 concentrations by ELISA

**Figure 1:**
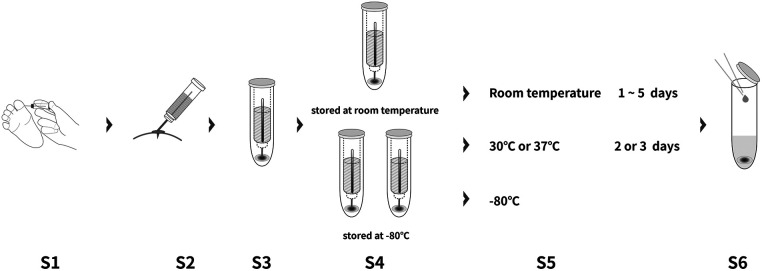
DBS device and process of sample collection and measurement.

150 μl of Calibrator Diluent (RD6-28) of the ELISA kit (R&D Systems) was added to the DBS tower. After soaking with shaking (Thermo-shaker BE-9008, Thermo Fisher Scientific, Waltham, MA, USA) for 1 h, 50 μl of the solvent extraction was loaded to detect the MMP-7 concentration on DBS. Serum and DBS MMP-7 concentration were measured by ELISA (R&D system, DMP700), following the manufacturer's protocol. Each sample was provided with three technical replicates, and the mean was recorded. All measurements were performed by WuXi Diagnostics (Shanghai, China), who were blinded to other test results and final diagnosis (Figure 1).

### Reference standard

Intraoperative cholangiography and subsequent histological examinations of liver biopsies were used to confirm BA diagnoses. Non-BA patients were confirmed by intraoperative cholangiography showing a patent biliary tree, and/or percutaneous transhepatic biopsy excluding BA, and/or genetic tests showing certain genetic mutations, and/or alleviation of symptoms without surgical intervention during follow-up for at least 3 months.

### Statistical analysis

Demographic and clinical characteristics of patients were summarized using conventional descriptive statistics, *n* (%) for categorical variables and median and quartiles (Q1, Q3) for continuous variables. Due to failing Shapiro-Wilk W test for normal distribution, between-group comparisons were performed using the Chi-squared and Mann–Whitney *U*-tests for all continuous variables. The consistency of DBS MMP-7 under different storage conditions was compared by Bland-Altman method. Linear regression was applied to assess the correlation of serum MMP-7 and DBS MMP-7 levels. The Delong test was performed to compare the accuracy between the two methods. Receiver operating characteristic (ROC) curves were constructed, and the area under the curve (AUC) and 95% confidence intervals (CIs) were reported as a measure of accuracy. The sensitivity, specificity, positive predictive value (PPV), and negative predictive value (NPV) were also used to show diagnostic accuracy. We used the maximum value of Youden's index as a criterion for selecting the optimal cut-off point. When the maximum value of Youden's index was achieved for multiple specificity values, the optimal cutoffs were defined based on achieving the maximum sensitivity.

A statistically significant difference was defined as a *P*-value <0.05. All data analyses were performed using R software 3.6.3 (R Foundation, Vienna, Austria). All authors had access to the study data and reviewed and approved the final manuscript.

## Results

### Study population

From December 2020 to October 2021, 241 infants with cholestasis undertook both serum and DBS MMP-7 measurement. 168 infants were diagnosed as biliary atresia through surgical exploration and subsequent histological examination (BA group). 73 infants were excluded of BA by intraoperative cholangiography showing a patent biliary tree, percutaneous transhepatic biopsy excluding BA, genetic tests showing certain genetic mutations, or alleviation of symptoms without surgical intervention during follow-up for at least three months (Non-BA group).

Demographic and baseline characteristics of the patients were summarized in Table 1. A majority of non-BA patients were male (68.5%), while the sex distribution was nearly equal in the BA group (46.4% male) (*P* = 0.002). The median age of the patients was 52 (IQR: 41, 68) days in the BA group and 75 (IQR: 49, 90) days in the Non-BA group (*P* < 0.001). The detailed description of other characteristics including TB, DB, GGT, ALT, AST were also listed in Table 1. As a result, GGT, TB and DB were identified to have significant differences between the BA and Non-BA groups (*P* < 0.05), whereas the two groups did not show any differences in ALT or AST (*P* > 0.05).

**Table 1:** Demographic and baseline characteristics of BA and Non-BA groups.

	BA(*n* = 168)	Non-BA(*n* = 73)	*P* *
Sex (male, %)	78 (46.4%)	50 (68.5%)	0.002
Age (days)^a^	52 (41, 68)	75 (49, 90)	<0.001
GGT (IU/l)^a^	406.7 (206.2, 657.2)	102.4 (64.2, 175.8)	<0.001
TB (umol/l)^a^	158.6 (125.9, 185.6)	130.8 (91.1, 182.9)	0.006
DB (umol/l)^a^	118.6 (95.6, 140.7)	95.8 (73.7, 130.8)	0.001
AST (IU/l)^a^	205.3 (133.2, 365.5)	203.2 (135.7, 374.0)	0.847
ALT (IU/l)^a^	129.4 (87.0, 228.7)	144.8 (88.4, 306.8)	0.144

BA, biliary atresia; GGT, gamma glutamyl transferase; AST, aspartate aminotransferase; ALT, alanine aminotransferase; TB, total bilirubin; DB, direct bilirubin.

^a^ Median (Q1, Q3).

* *P*-value between BA and Non-BA groups. Chi-square test were applied to gender, while the rest were tested by Mann–Whitney test.

### Correlation and consistency of serum and DBS MMP-7

**Figure 2:**
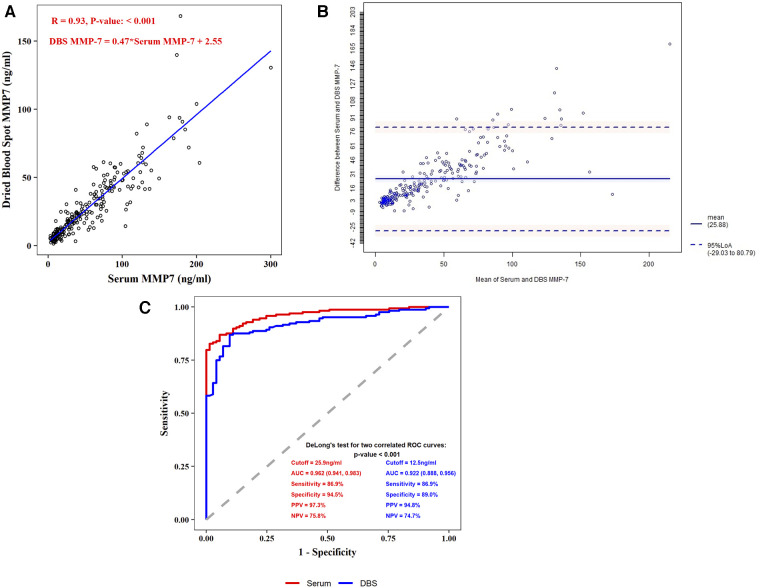
Correlation and comparison between serum MMP-7 and DBS MMP-7. (**A**) Spearman correlation coefficient test of serum and DBS MMP-7 (**B**) bland-altman consistency test of serum and DBS MMP-7 (**C**) receiver operating characteristic (ROC) plots for serum and DBS MMP-7.

Linear regression showed DBS MMP-7 correlated well with serum MMP-7 (*R* = 0.93, *P* < 0.001), and the regression formula was DBS MMP-7 = 0.47* Serum MMP-7 + 2.55 (Figure 2A). The Bland-Altman method showed that DBS MMP-7 was consistent with serum MMP-7 (95% values within 95% LoA) (Figure 2B).

### Diagnostic performance of serum and DBS MMP-7

In the BA group, the median concentration of serum MMP-7 was 64.25 ng/ml (IQR: 36.65, 94.13), and the median concentration of DBS MMP-7 stored at −80 °C was 30.38 ng/ml (IQR: 18.24, 47.57). In the Non-BA group, the median concentration of serum MMP-7 was 9.97 ng/ml (IQR: 7.34, 13.20), and the median concentration of DBS MMP-7 was 6.68 ng/ml (IQR: 4.86, 9.97). Further analysis showed both serum and DBS MMP-7 correlated with age in BA group, while the age has no significant correlation with MMP-7 levels in non-BA group (Supplementary Figures S1,S2). The best cut-off value of serum MMP-7 in the diagnosis of BA was 25.9 ng/ml, achieving the area under the ROC curve (AUC) of 0.962 (95% CI: 0.941, 0.983), and the sensitivity, specificity, positive predictive value (PPV), and negative predictive value (NPV) were 86.9%, 94.5%, 97.3% and 75.8%, respectively. The best cut-off value of DBS MMP-7 in the diagnosis of BA at −80 °C was 12.5 ng/ml, achieving the AUC of 0.922 (95% CI: 0.888, 0.956), and the sensitivity, specificity, PPV, and NPV were 86.9%, 89.0%, 94.8%, and 74.7%, respectively (Figure 2C).

### Thermal stability of DBS MMP-7

**Figure 3:**
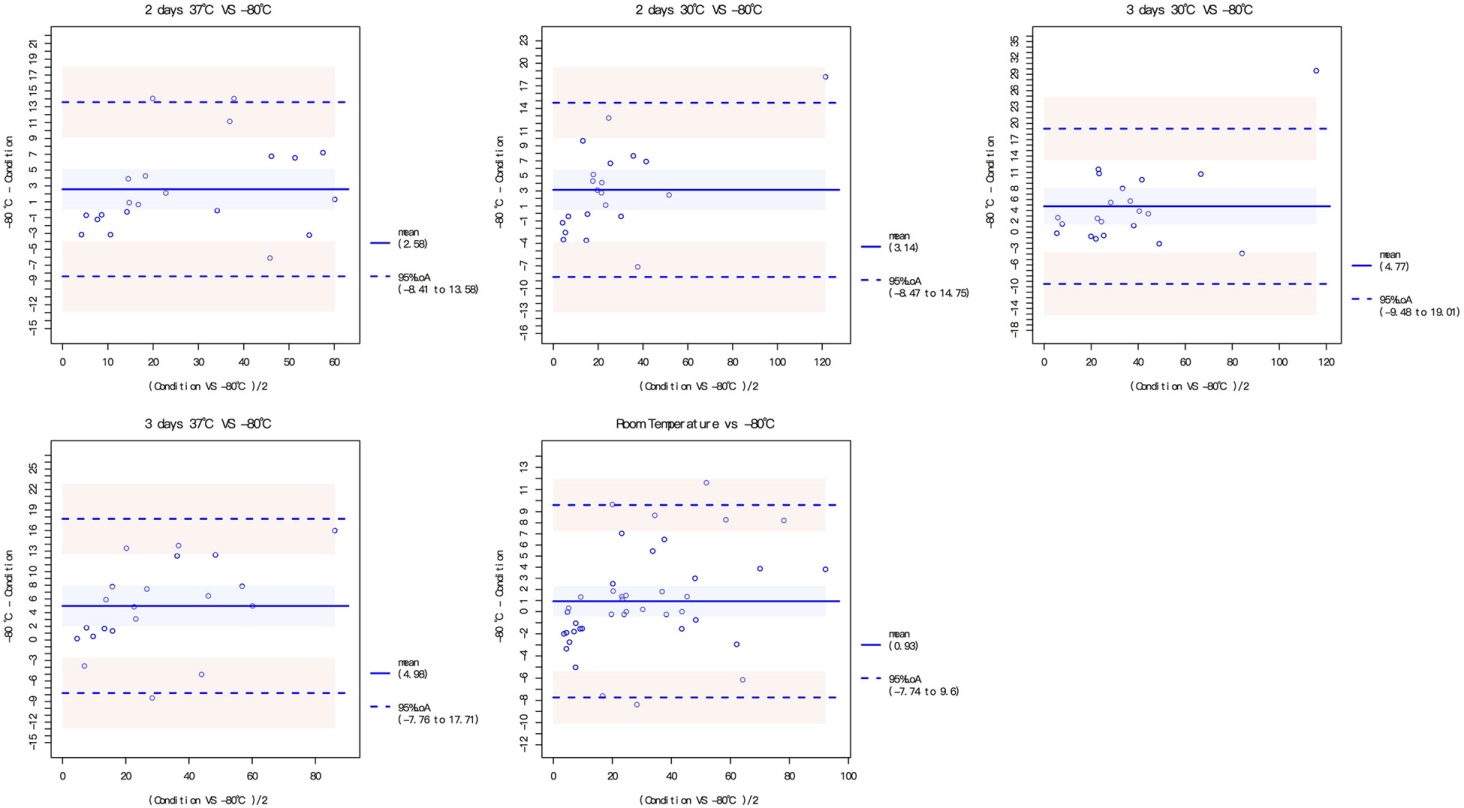
Consistency test for DBS under different storing conditions with that of −80 °C.

The dried blood spots were intervened under different storage conditions, including 1–5 days at room temperature, 2 or 3 days at 30 °C and 2 or 3 days at 37 °C. The DBS MMP-7 concentration under different storage conditions all had good correlation and consistency with that at −80 °C (Figure 3, Supplementary Table S1; Figure S3).

## Discussion

Accurately differentiating BA from other causes of non-BA cholestasis remains a challenge due to overlapping symptoms preoperatively. According to previous studies, serum MMP-7 holds great promise for discriminating BA (12–14).

This study aimed to develop an innovative approach of measuring MMP-7 using dried blood spot to collect micro-volume blood from heel tips, laying the foundation for its significant role in the diagnostic and screening algorithm for BA. We found that: (1) DBS MMP-7 levels correlated very well with serum MMP-7 levels; (2) DBS MMP-7 levels had a good accuracy in discriminating BA; (3) DBS MMP-7 had good thermal stability under different storage conditions.

This study further confirmed the promising diagnostic accuracy of MMP-7 as a biomarker for BA. To avoid investigator bias, all MMP-7 levels were measured by Wuxi Diagnostics, who were blinded to the clinical data. MMPs are involved in ECM remodeling, which is closely related to various physical and pathological processes, including liver fibrosis (16, 17). Biliary atresia is different from other cholestatic diseases in rapid fibrotic process in early stage. This might be why MMP-7 levels are significantly elevated in BA group.

In recent years, serum MMP-7 has been gradually integrated into diagnostic algorithm of BA during routine clinical practice in our center. However, fresh serum samples should be applied for serum MMP-7 measurements in case of protein degradation, leading to high requirements for sample collection and storage. Besides, only 10–20 µl of serum is required for ELISAs, resulting in unnecessary blood waste.

Thus, we developed a noninvasive method to collect heel tip blood, which can easily be used to detect MMP-7 concentrations via DBS. The DBS device in this study is different from standard DBS. It comprises a quantificational collection vessel, a sample collection tube and a sample collection filter paper. The quantificational collection vessel is a 10-μl capillary tube, which is necessary for DBS MMP-7 measurement. The sample collection tube helped to separate the DBS from outside environment to facilitate its transportation. The filter paper is made of glass fiber and attached with calcium chloride, which can shorten the drying time after blood sample collection. It can effectively absorb whole blood including red blood cells. However, the standard DBS is usually made of cotton fiber and is not quantificational for blood collection. A more recent study using standard DBS has confirmed the screening performance of MMP-7 as early as 3 days old (18). The novel quantificational DBS device in this study may provide a more accurate measurement.

DBS and serum MMP-7 couldn't be equivalent because there is a matrix effect. That is, the serum accounts for about half of the whole blood, and that's why the slope of relationship between DBS and serum MMP-7 is around 0.5. DBS MMP-7 correlated well with serum MMP-7, with R of 0.93. DBS MMP-7 has good accuracy for diagnosing BA, though serum MMP-7 showed better accuracy. However, DBS is less invasive given the need for only 10 μl of blood. Thermal stability showed that it is convenient to be transported at room temperature. Thus, it could be possible to add BA screening to the myriad tests performed on neonatal heel blood screening and for parents to collect the samples themselves at home and mail them to an institution for measurement.

This study has some remaining limitations. First, the BA and non-BA groups in our study were not age-matched because many of the non-BA patients had been first referred to local hospitals, thereby delaying the first visit to our hospital. However, age showed no significant correlation with both DBS and serum MMP-7 levels in the non-BA group; thus, the chance of induced bias was small. Second, subgroup analysis of neonates was not performed since the sample size was small. Further study is warranted to validate the diagnostic accuracy of serum and DBS MMP-7 before its application in neonates screening. Third, operator bias may exist because this was a single center study and the DBS samples were all collected by the same investigator (JJ). It may take some time to have a command of DBS collection since quantification is necessary for DBS MMP-7 measurement. Thus, the standardized protocol of the use of DBS collection device should be made before its clinical application and multicenter studies are needed to further validate its consistency and accuracy.

## Data Availability

The raw data supporting the conclusions of this article will be made available by the authors, without undue reservation.
