# Supplementary material for: Measurement of MMP-7 in micro-volume peripheral blood: development of dried blood spot approach
Source: Front Pediatr. 2023 Nov 15;11:1293329. doi: 10.3389/fped.2023.1293329 (PMC10684727; doi:10.3389/fped.2023.1293329)
Supplement: Supplementary file 1 [file Datasheet1.pdf]

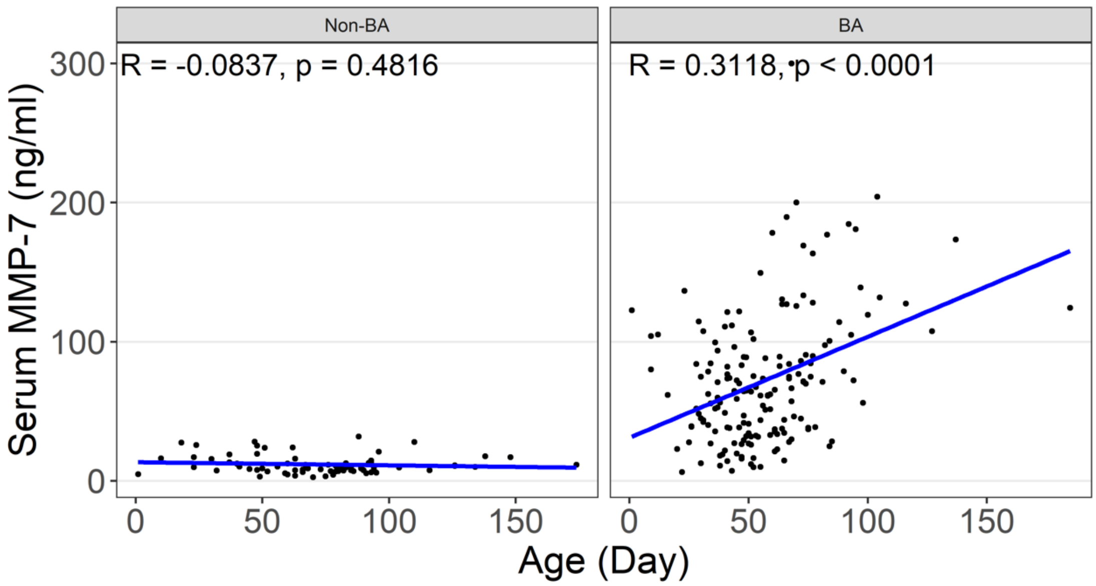

Figure S1. Correlation of serum MMP-7 and age. Serum MMP-7 was correlated to age only in BA group. The R and P were shown.

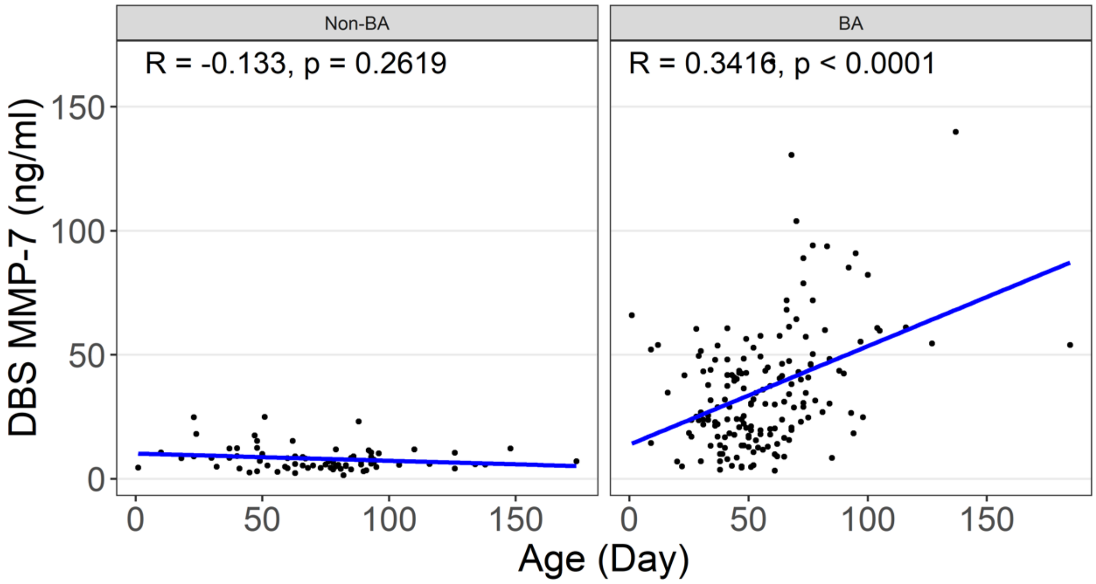

Figure S2. Correlation of DBS MMP-7 and age. DBS MMP-7 was correlated to age only in BA group. The R and P were shown.

Table S1: Thermal stability test of DBS

Storage temperature (℃)	Days of storage (days)	N	Median (IQR)	-80 °C Median (IQR)	Correlation (R)[a]	Consistency[b]
Room temperature	1-5	43	23.9 (9.9, 44.0)	24.7 (8.7, 43.2)	0.97	0.93
30	2	21	18.3 (15.3, 30.4)	22.8 (15.2, 31.0)	0.95	0.95
37	2	21	16.4 (12.6, 42.8)	23.9 (14.1, 44.9)	0.95	0.90
30	3	21	25.6 (20.3, 38.5)	31.0 (24.0, 46.0)	0.93	0.95
37	3	21	21.6 (11.8, 42.1)	25.0 (16.5, 43.6)	0.93	0.95

[a]: Spearman correlation coefficient test was applied.

[b]: The Bland-Altman method was applied.

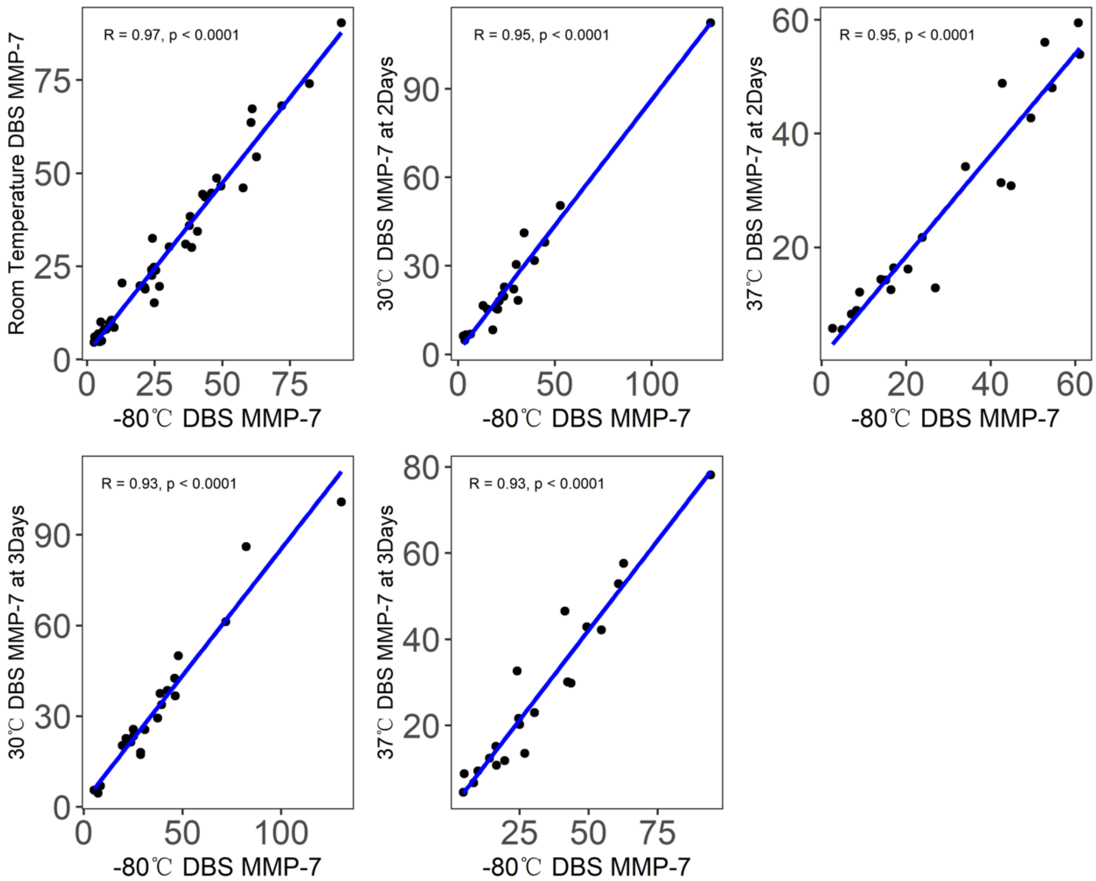

Figure S3. Correlations analysis for DBS under different storing conditions with that of -80℃
